# Green and novel cleaning method by plasma-activated water on a microfiltration membrane after apple juice clarification

**DOI:** 10.1016/j.fochx.2026.104317

**Published:** 2026-08-17

**Authors:** Najmeh Ghahari, Yousef Ramezan, Hossein Mirsaeedghazi, Alireza Faraji, Shahrooz Saviz

**Affiliations:** a Department of Food Science and Technology, TeMS. C., Islamic Azad University, Tehran, Iran; b Nutrition & Food Sciences Research Center, TeMS. C., Islamic Azad University, Tehran, Iran; c Department of Food Technology, Faculty of Agricultural Technology, College of Agriculture and Natural Resources, University of Tehran, Tehran, Iran; d Department of Organic Chemistry, TeMS. C., Islamic Azad University, Tehran, Iran; e Plasma Physics Research Center, Science and Research Branch, Islamic Azad University, Tehran, Iran

**Keywords:** Plasma-activated water, Juice clarification, Mixed cellulose esters, Microfiltration, Membrane recovery

## Abstract

This study presents the first systematic optimization of plasma-activated water (PAW) generation parameters for cleaning fouled plasma-modified mixed cellulose ester (MCE) microfiltration membranes employed in apple juice clarification. The effects of cold plasma (CP) water activation time (5, 7, and 9 min) and electrode-to-water surface distance (20, 30, and 40 mm) on permeate flux, PAW pH, and the maximum number of effective cleaning cycles were optimized using Response Surface Methodology (RSM) to evaluate the impacts of these independent variables and their interactions simultaneously, developing predictive models with fewer experimental runs. Statistical analysis revealed that activation time, electrode-to-water surface distance, and certain interactions significantly affected all response variables (*P* *<* *0.05*). Flux recovery ratio (FRR) identified a 9 min activation time and a 30 mm electrode-to-water surface distance as the optimal conditions. Unlike previous studies focused primarily on microbial inactivation and food decontamination, the present study establishes optimized PAW generation conditions for sustainable membrane cleaning and enhanced membrane reusability, considering the limited efficacy of water-only cleaning and the adverse effects of chemical cleaning to reduce chemical consumption and membrane degradation while promoting more novel and greener membrane-based separation processes in the food industry.

## Highlights

• Plasma-activated water (PAW) has been used for membrane cleaning.
• The membrane and flux recovery improved after the cleaning procedure.
• PAW washing preserved membrane pore morphology better than chemical washing.
• Washing the membrane with PAW did not change the color parameters of apple juice.

## Graphical abstract

**Figure:**
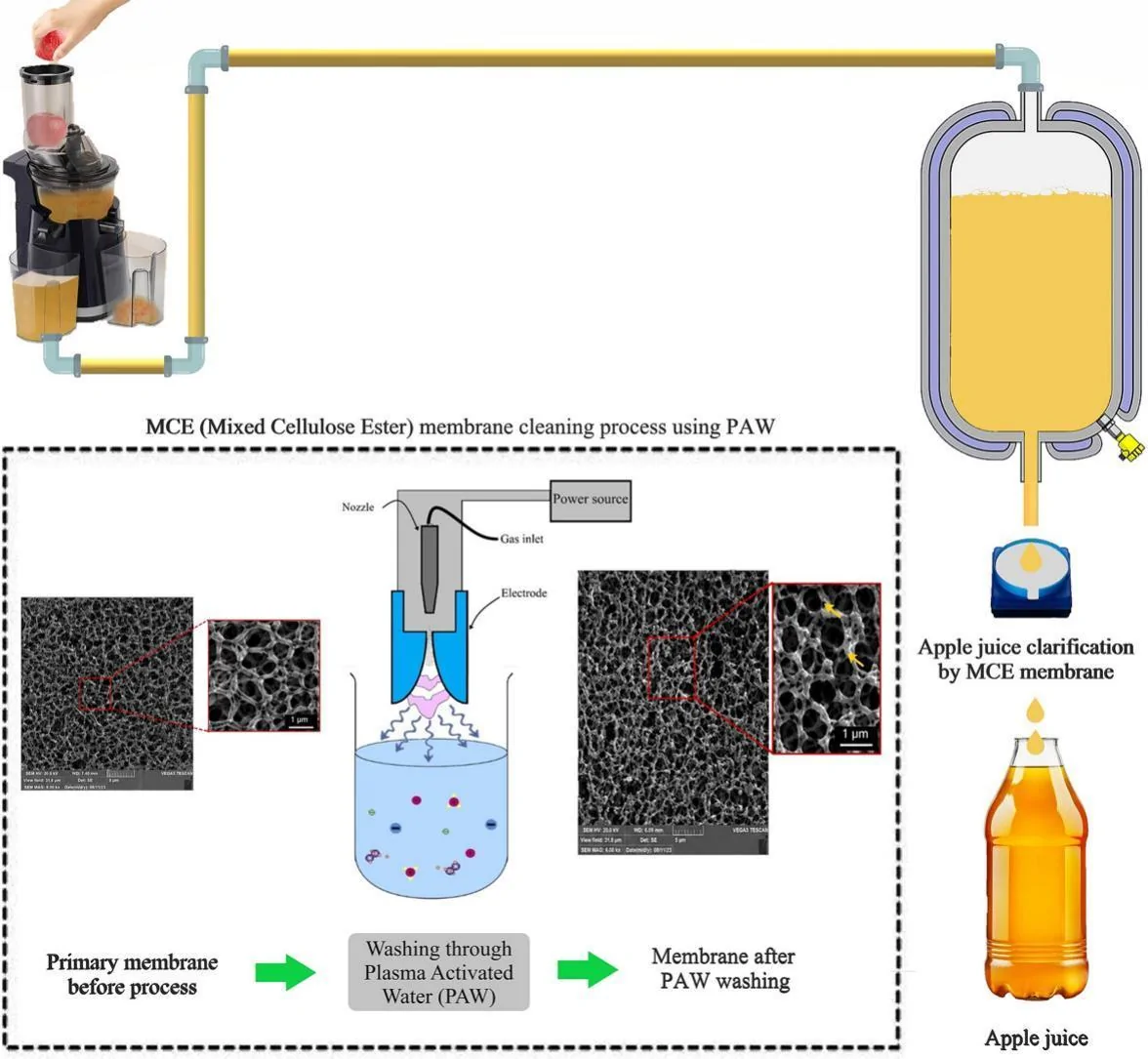

## 1 Introduction

Apple juice clarification is performed to remove suspended solids, pectin, and other haze-forming substances, yielding a bright and transparent juice. The process generally involves a combination of enzymatic treatment, filtration, and, occasionally, the use of fining agents or advanced physical technologies. While clarification improves the visual appearance of apple juice, it also decreases the concentrations of health-promoting polyphenols and antioxidant compounds compared with cloudy juice (Dussling et al., 2025; Kereszturi et al., 2025; Zavarise et al., 2025). Advanced and novel clarification strategies and processing aids continue to be developed to optimize both clarity and nutritional quality during the production of apple juice. Among these, membrane filtration has emerged as a promising technology for apple juice clarification (Elshafei et al., 2025; Ghahari, Ramezan, et al., 2024). Microfiltration membranes, in particular, are widely employed to remove pectin, suspended solids, and colloidal particles while preserving nutritional and sensory qualities of the juice. Compared with conventional enzymatic and thermal clarification processes, microfiltration delivers several advantages, including higher yield and improved retention of natural color and aroma (Vilar et al., 2025). However, membrane fouling remains a major challenge in juice filtration, as it reduces permeate flux and compromises overall process efficiency. Fouling occurs when juice components accumulate on the membrane surface or within its pores, resulting in pore blockage, higher filtration resistance, and reduced membrane performance (Al-Kathiri et al., 2025). Various strategies have been explored to mitigate membrane fouling during juice filtration. For instance, the application of rotating blades generates shear forces that reduce concentration polarization and cake formation, thereby enhancing permeate flux (Yassari et al., 2023). Moreover, surface modification techniques, such as low-pressure oxygen plasma treatment, have also shown the capability to improve membrane hydrophilicity and reduce foulant adhesion (Ghahari, Mirsaeedghazi, et al., 2024; Katibi et al., 2023). Additionally, the application of magnetic or electric fields can decrease membrane fouling during juice clarification (Motaghian et al., 2025). To mitigate membrane fouling, maintain filtration performance, preserve juice quality, and reduce operational costs during membrane-based fruit juice clarification, effective membrane recovery and cleaning protocols are essential. Such protocols play a critical role in restoring membrane functionality and prolonging membrane durability by integrating physical and chemical cleaning approaches to remove both organic foulants, such as pectin and proteins, and inorganic deposits, such as calcium-based scales (Abd-Razak et al., 2021; Katibi et al., 2023; Rajendran et al., 2025). Common physical recovery techniques include permeate flow reversal to detach surface-deposited foulants, air bubble injection to enhance hydrodynamic turbulence and reduce cake layer formation, and high-frequency ultrasonic treatment to disrupt fouling layers (Chung et al., 2023; Wahia et al., 2024).

In parallel, chemical cleaning strategies, including alkaline treatment for the removal of organic foulants and acidic cleaning for the dissolution of inorganic scales (e.g., calcium oxalate), are widely employed while minimizing potential membrane damage. In appropriately optimized conditions, the integration of these approaches can achieve high flux recovery rates (85–95%) and reduce energy consumption by 20–30% (Conidi et al., 2020; Shahbazi et al., 2021). Despite their effectiveness, the repeated, long-term application of conventional physical and chemical cleaning methods can lead to membrane degradation, chemical residues, and environmental concerns, highlighting the need for more sustainable membrane-cleaning strategies. Although various approaches, including alternating alkaline and acidic cleaning cycles, backwashing, ultrasonic cleaning, air scouring, and membrane surface modification, have been extensively investigated, their long-term practical application remains limited by membrane aging, incomplete foulant removal, operational complexity, and environmental impacts (Chung et al., 2023; Shahbazi et al., 2021; Wahia et al., 2024). Collectively, these limitations necessitate more effective, eco-friendly, and sustainable cleaning alternatives that maintain membrane function while reducing chemical dependency and operational complexity on an industrial scale.

Plasma technology has demonstrated significant potential in food processing applications, including microbial decontamination and shelf-life extension, due to the generation of reactive species capable of degrading organic contaminants (Shirazi et al., 2025; Singh et al., 2025). Although plasma-based membrane modification has been successfully applied to improve membrane performance during apple juice clarification by reducing fouling and increasing selectivity, the application of plasma technology for membrane cleaning remains an emerging approach that has yet to be established in juice processing (Ghahari, Mirsaeedghazi, et al., 2024; Ghahari, Ramezan, et al., 2024). MCE membranes are widely used in fruit juice clarification due to their biocompatibility, relatively high permeate flux, and ability to preserve juice quality. Previous research demonstrated that plasma modification of MCE membranes significantly enhanced apple juice clarification performance by improving membrane hydrophilicity and reducing membrane fouling compared with unmodified membranes (Ghahari, Mirsaeedghazi, et al., 2024; Ghahari, Ramezan, et al., 2024). Therefore, plasma-modified MCE membranes were employed to evaluate the effectiveness of PAW as a membrane cleaning and recovery strategy under optimized membrane conditions. PAW is an indirect form of CP technology in which deionized water is exposed to plasma, leading to the formation of reactive oxygen and nitrogen species (RONS). Consequently, as the plasma exposure time increases, the pH of PAW decreases due to the generation of such acidic reactive species. This acidification can be mainly attributed to the formation of nitric acid (HNO_3_) and peroxynitrous acid (ONOOH) through reactions involving nitrogen oxides (NO, NO_2_, and NO_3_), as well as the production of hydronium ions (H_3_O^+^) related to hydrogen peroxide (H_2_O_2_)-mediated reactions (Jafari et al., 2025). Studies have shown the potential of PAW in a variety of membrane-related applications, including wastewater treatment, biofilm inactivation, desalination systems, and antimicrobial cleaning, primarily through the function of RONS, which enable microbial inactivation, organic pollutants degradation, and biofouling mitigation (Mazandarani et al., 2025; Wang et al., 2024). PAW has delivered considerable potential in removing contaminants and reducing membrane fouling due to its strong oxidative capacity and eco-friendly nature. However, its application for the recovery of fouled microfiltration membranes used in fruit juice clarification has not been investigated. In contrast to desalination and wastewater treatment membranes, fouling during apple juice clarification is mainly caused by the accumulation of pectin, polysaccharides, proteins, and phenolic compounds, which present distinct challenges and require different cleaning mechanisms and operational strategies (Li et al., 2022). PAW has emerged as a promising and eco-friendly alternative to conventional chemical cleaning agents for membrane cleaning and biofouling control owing to its safe nature. However, no previous research has optimized PAW generation parameters for the recovery of fouled microfiltration membranes used in apple juice clarification. Therefore, the present study aimed to optimize PAW generation conditions for the first time to maximize membrane recovery cleaning efficiency and recovery, restoring membrane performance and extending its reusability during the apple juice clarification process using RSM. Compared with conventional statistical and experimental methods, RSM enables the simultaneous evaluation of multiple variables and their interactions, facilitates the development of predictive models, determines the optimal operating conditions, and significantly reduces the experimental runs. RSM, therefore, delivers a time-efficient framework for accurate and reliable optimization. This study provides a scientific foundation for developing sustainable membrane cleaning protocols. Also, its findings can facilitate the broader adoption of novel technologies, such as CP and especially PAW, in juice clarification and other separation processes in the food industry.

## 2 Materials and methods

### 2.1 Juice preparation

Fresh Golden Delicious apples were purchased from a local market (Taleghan, Iran). The ripe fruits were thoroughly washed, and the juice was extracted using a centrifugal juicer (JE1100, Kenwood, England). The extracted juice was then filtered through a No. 9 mesh sieve (2 mm) to remove the apple pulp.

### 2.2 Microfiltration of apple juice

A cross-flow membrane filtration system equipped with a 2-L feed tank was used. For each experiment, 1.5 L of apple juice was loaded into the feed tank and continuously recirculated through the membrane module. Due to the cross-flow recirculation mode operation of the system, the feed volume remained approximately constant during filtration, and the effective processed juice volume was considered as the initial feed volume (1.5 L). Based on previous research, a CP-treated MCE membrane (Qingfeng Filter Equipment Co., effective surface area of 0.0098 m^2^, pore size of 0.22 μm, molecular weight cut-off (MWCO) <30 kDa, and an initial pure water flux of ≥25 mL/min.cm^2^ at 25 °C, and 100 kPa) was applied for apple juice clarification. The membrane was treated for 180 s with a 10-W plasma using air as the working gas, 60 kPa feed pressure, and 15 mL/s flow rate at 40 °C (Ghahari, Mirsaeedghazi, et al., 2024; Ghahari, Ramezan, et al., 2024). A flat-sheet cross-flow membrane module equipped with a rotary vane pump (Fluid-o-Tech, Italy) was employed to circulate the feed solution at a constant flow rate, while hydrodynamic pressure drove the separation process. A WIKA S-10-3 A pressure transmitter (WIKA, USA) coupled with an inverter (Delta VFD-M, Delta Products Corporation, USA) was used to regulate the feed flow rate and transmembrane pressure. The permeate weight, as the primary output parameter of microfiltration, was continuously recorded by measuring its change over time (Δt). The permeate flux (JP) was calculated according to Eq. (1).

(1) $$J_{p}=\frac{∆m}{A.t}$$

Where: Δm, A, and t represent the permeate weight (kg), the effective membrane surface area (m^2^), and the operation time (s), respectively.

### 2.3 PAW generation

**Fig. 1:**
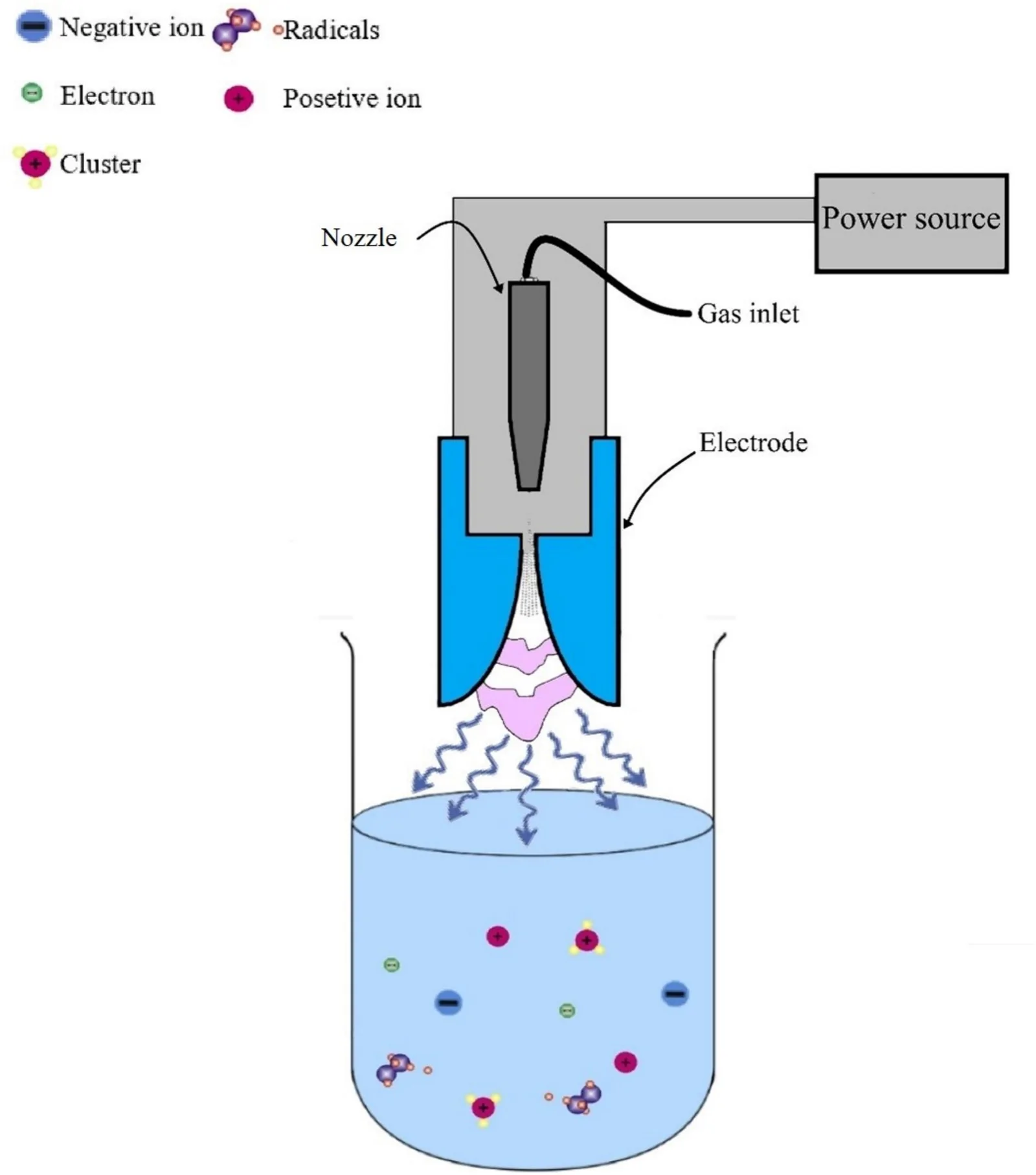
Schematic of the Lab-scale plasma-activated water production setup.

PAW for membrane recovery was generated using an AC-powered gliding arc plasma system (Plasma Clean - Satia Company, Iran) operating at a voltage of 15 kV and a frequency of 50 Hz under atmospheric pressure, with air as the working gas for reactive species generation (Fig. 1). The distance between the electrode tip and the water surface was set to 20, 30, and 40 mm. The deionized water was exposed and activated for 5, 7, and 9 min (Damough et al., 2025). The discharge power was not directly measured. PAW was used immediately after its generation. No storage period was applied before membrane cleaning to minimize the loss of reactive species and maintain PAW's cleaning efficacy.

### 2.4 Experimental design

A series of preliminary experiments was conducted to determine the optimal gliding arc plasma conditions for producing PAW that maximized permeate flux during fouled membrane recovery. The factors included activation time (5, 7, and 9 min) and the electrode-to-water surface distance (20, 30, and 40 mm). Due to the dependence of the composition and concentration of reactive species in PAW on the plasma operating conditions, the effects of these variables on permeate flux during apple juice clarification were evaluated using RSM, as shown in Table 1. Then, using Eq. (2), a regression model describing permeate flux was subsequently developed. The predictive performance of the model was evaluated using the coefficient of determination (R^2^).

(2) $$Y=\beta_{0}+\sum \beta_{i}X_{i}+\sum \beta_{\mathrm{ij}}X_{i}X_{j}+\sum \alpha_{i}X_{i}^{2}$$

**Table 1:** Evaluated pH of PAW and the maximum number of cleaning cycles before membrane aging at each designed experiment (CCD design).⁎

Run	Time (min)	Distance (mm)	pH	Permeate flux after first cleaning process (kg/ (m^2^.h))	Maximum number of cleaning cycles
1	9	30	3.36 ± 0.04	6.54 ± 0.15	6.0 ± 0.0
2	5	40	3.98 ± 0.05	4.78 ± 0.18	3.0 ± 0.0
3	7	20	3.18 ± 0.03	3.45 ± 0.16	2.0 ± 0.0
4	7	30	3.46 ± 0.04	5.43 ± 0.07	5.0 ± 0.0
5	9	20	2.85 ± 0.05	3.03 ± 0.14	2.0 ± 0.0
6	7	30	3.49 ± 0.04	5.43 ± 0.17	5.0 ± 0.0
7	5	30	3.87 ± 0.05	4.69 ± 0.06	4.0 ± 0.0
8	7	30	3.50 ± 0.04	5.47 ± 0.18	5.0 ± 0.0
9	7	40	3.61 ± 0.05	4.72 ± 0.17	3.0 ± 0.0
10	9	40	3.40 ± 0.04	5.64 ± 0.16	5.0 ± 0.0
11	5	20	3.56 ± 0.05	3.09 ± 0.10	3.0 ± 0.0

⁎ Values are presented as mean ± SD (*n* = 3) from independent experimental replicates.

Where: Y, X, $\beta_{0}$,$\;\beta_{i}$,$\;\beta_{\mathrm{ij}}$, and $\alpha_{i}$ represent the predicted response, actual values of each factor, constant, and coefficients of the presented model, respectively.

The significant regression models for predicting the pH of PAW, permeate flux after the first cleaning process, and the maximum number of cleaning cycles before membrane aging, based on the actual levels of the process variables, are presented in Eq. (3).

(3) $$Y=\beta_{0}+\beta_{1}\times \mathrm{Time}+\beta_{2}\times \mathrm{Distance}+\beta_{12}\times \mathrm{Time}\times \mathrm{Distance}+\beta_{11}\times {\left(\mathrm{Time}\right)}^{2}+\beta_{22}\times {\left(\mathrm{Distance}\right)}^{2}$$

Where: *β*_*0*_, *β*_*1,*_ *β*_*2,*_ *β*_*12,*_ *β*_*11,*_ and *β*_*22*_ represent the coefficients of the model (Table 5). The accuracy of the model-predicted response was expressed by the coefficient of determination (R^2^).

### 2.5 Cleaning procedure and FRR

During apple juice microfiltration, permeate flux gradually declined due to foulant deposition on the membrane surface and within its pores, reaching a nearly steady-state value after approximately 60 min. Therefore, a filtration time of 60 min was used to obtain a fouled membrane before each cleaning. After fouling, the membrane was chemically cleaned using sequential alkaline and acidic treatments to remove organic, microbial, and mineral foulants and restore membrane performance. The membrane was first cleaned with a 0.5% (*w*/*v*) alkaline solution at approximately 80 °C for 30 min, followed by rinsing with deionized water until a neutral pH was reached. Subsequently, the membrane was treated with a 0.1% (w/v) HCl solution for 30 min to remove mineral scale. Then, the membrane was rinsed again with deionized water to eliminate residual acid and restore a neutral membrane environment before reuse (Shahbazi et al., 2021). Before PAW cleaning, the fouled membrane was rinsed with deionized water to remove loosely attached foulants and facilitate contact with PAW. For each PAW cleaning, 1.5 L of freshly prepared PAW was circulated through the membrane module for 30 min at ambient temperature (approximately 27 °C) under the same hydrodynamic conditions as the filtration experiments (feed flow rate of 15 mL/s, transmembrane pressure of 60 kPa, and cross-flow configuration). After treatment, the membrane was thoroughly rinsed with deionized water to remove residual reactive species and detached foulants. Membranes fouled during apple juice clarification were cleaned using PAW generated under different plasma operating conditions and, for comparison, by the conventional alkaline-acid cleaning procedure. Cleaning efficiency was evaluated by comparing the recovered permeate flux after cleaning with the initial permeate flux before membrane cleaning. The membrane recovery cycles are evaluated by measuring permeate flux after each cleaning procedure. The effectiveness of membrane cleaning was quantified using the FRR, which is based on permeate flux measurements. The cleaning performance of PAW-treated membranes was compared with that of the conventional alkaline-acid cleaning procedure (0.5% *w*/*v* NaOH followed by 0.1% w/v HCl), which served as the control treatment. The permeate flux of the clean membrane before fouling was used as the reference value for FRR calculations. To assess the long-term cleaning performance and membrane reusability, repeated fouling-cleaning cycles were conducted. For multiple cleaning cycles (n), the FRR was determined using Eq. (4).

(4) $${\mathrm{FRR}}_{n}=\frac{J_{\text{after}\;n_{\mathrm{th}}\;\text{cleaning}}}{J_{\text{clean membrane}}}\times 100$$

Where: J_after n_^th^_cleaning_ represents the permeate flux measured after the nth cleaning cycle of the fouled membrane, and J_clean membrane_ expresses the permeate flux of the membrane when new or fully cleaned before fouling. The maximum number of cleaning cycles was defined as the cycle at which the FRR decreased below the predetermined acceptance threshold of 60%, indicating incomplete foulant removal or progressive membrane aging.

### 2.6 Membrane and clarified apple juice evaluation

The cross-sectional morphology of the membranes before and after the first recovery was evaluated by scanning electron microscopy (SEM) (TESCAN VEGA3, Czech Republic) operated at an accelerating voltage of 20.0 kV. Membrane samples were cryogenically fractured in liquid nitrogen to obtain clean cross-sections and subsequently sputter-coated with a conductive gold layer before the SEM analysis (Ghahari, Mirsaeedghazi, et al., 2024). The membrane color parameters (L* a* b* values) were measured using a HunterLab colorimeter (Gulec et al., 2017).

### 2.7 Statistical analysis

A central composite design (CCD) was implemented using Design-Expert software (version 7.0) to optimize the plasma operation conditions. The permeate flux after the first cleaning cycle, the pH of PAW, and the maximum number of cleaning cycles before membrane aging were selected as response variables. The maximum number of cleaning cycles was treated as an ordinal performance indicator of membrane cleaning durability and incorporated into the RSM framework for comparative optimization. All experiments were performed independently in triplicate under identical operating conditions to ensure reliability and reproducibility of the results. Analysis of variance (ANOVA) was conducted to evaluate the significance of the experimental factors and their interactions, and polynomial regression equations along with three-dimensional response surface plots were generated using Design-Expert software. The color parameters of permeate and retentate obtained during apple juice clarification were analyzed by one-way ANOVA followed by Duncan's multiple range test using the SPSS software (Version 18.0; SPSS Inc., Chicago, IL, USA). All color measurements were performed in triplicate.

## 3 Results and discussion

The permeate flux after the first cleaning cycle, the pH of PAW, and the maximum number of cleaning cycles before membrane aging were selected as responses for each experimental run, summarized in Table 1. Statistical analysis was performed to evaluate the effect of the PAW activation time and the electrode-to-water surface distance on each response.

### 3.1 pH of PAW

**Fig. 2:**
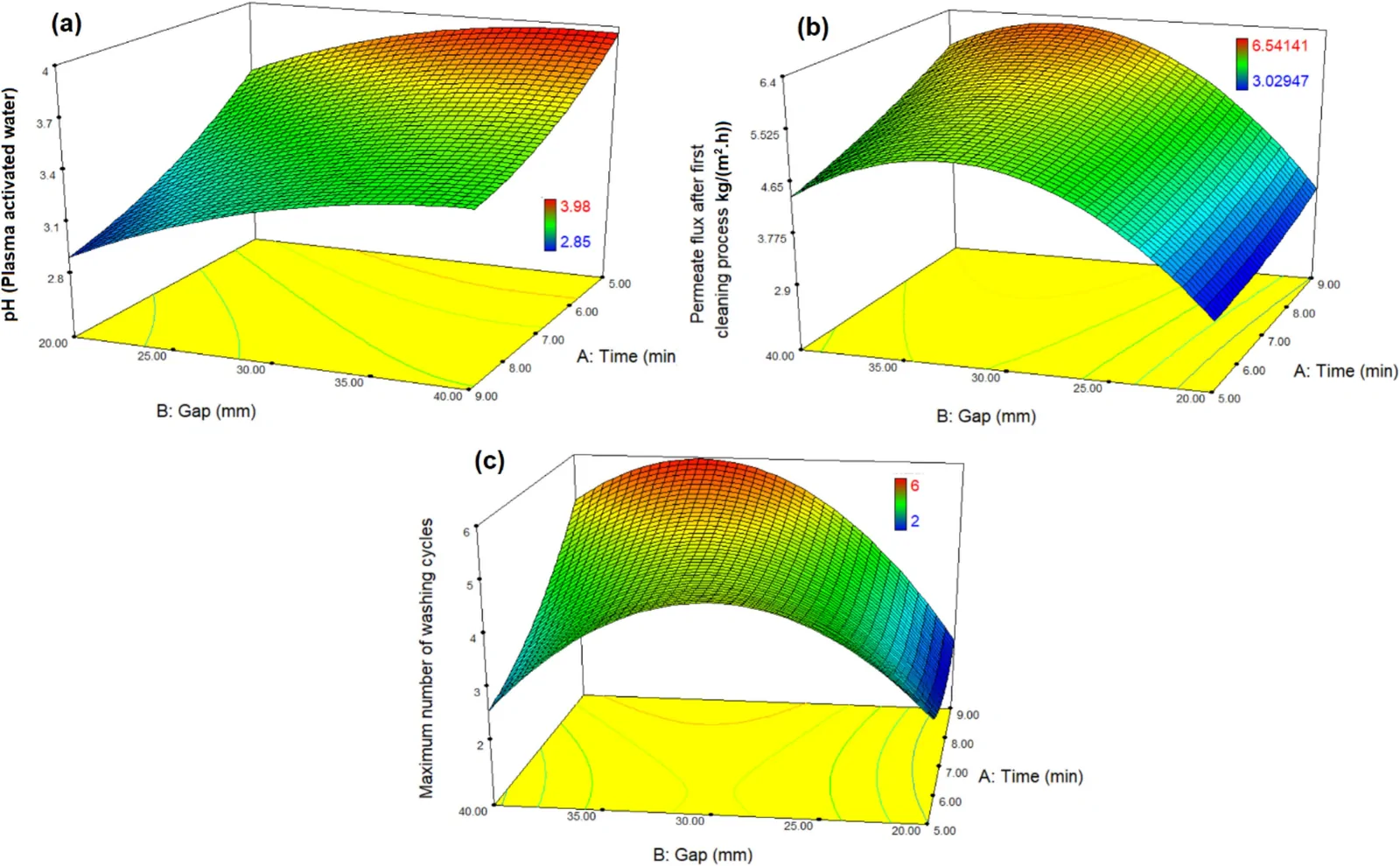
The effect of water activation time with CP (A) and electrode-to- water surface distance (B) of PAW production on a) pH of PAW, b) Permeate flux after first cleaning, c) Number of cleaning cycles in the membrane process of apple juice clarification.

As shown in Table 2, the gliding arc plasma operating parameters significantly affected the pH of PAW (*P* *<* *0.05*). Analysis of the interaction effects revealed the significant effects of quadratic terms (A^2^ and B^2^) on the pH of PAW (*P* *<* *0.05*), along with no significant effect of the two-factor interaction term (AB) on the pH of PAW (*P* *>* *0.05*). The pH of PAW decreased by approximately 2.76–28.39% as activation time increased from 5 to 9 min and the distance between the electrodes and the water surface decreased from 40 to 20 mm (Fig. 2a). This acidification can be primarily linked to the enhanced generation of reactive species during plasma treatment, which promotes the formation of acidic compounds, including hydrogen peroxide, nitrates, and nitrites. The higher reduction in pH observed with longer activation times and shorter electrode-to-water distances can be described by the intensified plasma-liquid interactions, increasing the production and dissolution of reactive species (Jafari et al., 2025). Plasma treatment can reduce the pH of water from near-neutral values (approximately 7) to highly acidic conditions (2.5–4.0), with longer exposure durations producing a more rapid and pronounced decline in pH (Bohlooli et al., 2024).

**Table 2:** ANOVA for the effect of the gliding arc plasma parameters on pH of PAW.

Source	Sum of Squares	df	Mean Square	F Value	*P*-value
Model	0.92	5	0.18	77.43	0.0001
A (Plasma Time)	0.54	1	0.54	226.73	0.0001
B (Distance)	0.33	1	0.33	137.16	0.0001
AB	0.0042	1	0.0042	1.77	0.2404
A^2^	0.021	1	0.021	8.62	0.0324
B^2^	0.043	1	0.043	17.98	0.0082
Lack of Fit	0.011	3	0.0036	…	….

### 3.2 Permeate flux

ANOVA of the PAW production parameters on membrane recovery, as evaluated by the permeate flux after the first cleaning process, is presented in Table 3. The results indicate that both gliding arc plasma operating parameters, namely activation time (A) and electrode-to-water surface distance (B), significantly affected the permeate flux following the first cleaning cycle (*P* *<* *0.05*). Among the interaction terms, only the quadratic effect of the electrode-to-water surface distance (B^2^) was statistically significant, indicating a nonlinear relationship between this parameter and membrane recovery (*P* *<* *0.05*). On the contrary, the remaining interactions showed no statistically significant effect on the permeate flux after the first cleaning process (*P* *>* *0.05*). As shown in Fig. 2b, the highest permeate flux was obtained when PAW was generated using a 9-min activation time and a 30 mm electrode-to-water surface distance. In contrast, the lowest permeate flux was observed at the same activation time with a reduced electrode-to-water surface distance of 20 mm. Extending the plasma activation time improves the generation of reactive species, including radicals and ions, in the water, thereby intensifying the treatment impacts and promoting the oxidation and chemical degradation of membrane foulants (Burlica et al., 2006).

**Table 3:** ANOVA for the effect of PAW production parameters for membrane recovery on the permeate flux.

Source	Sum of Squares	df	Mean Square	F Value	P-value
Model	11.93	5	2.39	13.05	0.006
A (Plasma Time)	1.29	1	1.29	7.05	0.045
B (Distance)	5.15	1	5.15	28.15	0.003
AB	0.16	1	0.16	0.86	0.395
A^2^	0.029	1	0.029	0.16	0.707
B^2^	5.13	1	5.13	28.08	0.003
Lack of Fit	0.91	3	0.30	…	…

Cleaning membranes with PAW can improve membrane hydrophilicity, enhancing membrane wettability and contributing to permeate flux recovery. Such an enhancement can be associated with the RONS functions generated during the CP treatment, which can modify the physicochemical properties of the membrane surface and interact indirectly with the fouling layer. However, in the absence of direct surface characterization, any changes in membrane surface chemistry or chemical functionalization remain speculative and cannot be conclusively confirmed. The fouling layer formed during apple juice clarification is primarily composed of pectin, starch, proteins, suspended solids, and phenolic compounds that accumulate on the membrane surface during the filtration process. Among these particles, pectin and starch readily interact to form a compact gel-like network, particularly under elevated temperatures, resulting in increased hydraulic resistance and a substantial decline in permeate flux. Membrane hydrophilicity plays a pivotal role in mitigating the deposition of such foulants, as hydrophilic surfaces exhibit a lower affinity for high-molecular-weight organic compounds. Consequently, enhanced hydrophilicity promotes water transport while reducing interfacial interactions between the membrane surface and organic foulants, thereby suppressing fouling development. In addition, PAW can facilitate membrane cleaning through the oxidative activity of RONS, which can react with the organic components of the fouling layer, promoting the oxidation, fragmentation, and partial degradation of complex organic macromolecules, weakening the structural integrity and adhesion of the fouling layer to the membrane surface (Chen et al., 2022; Egorova et al., 2024; Mai-Prochnow et al., 2021; Shah et al., 2024).

From a mechanistic perspective, the cleaning efficacy of PAW can be attributed to the synergistic action of RONS, including hydroxyl radicals (^•^OH), hydrogen peroxide (H_2_O_2_), nitrite (NO_2_^−^), and nitrate (NO_3_^−^) species. Among the reactive species identified in PAW systems, H_2_O_2_ and nitrate/nitrite species are considered relatively long-lived and are therefore capable of sustaining oxidative activity during the membrane cleaning process. H_2_O_2_ can penetrate the fouling layer and induce oxidative degradation of organic compounds, such as pectin, proteins, and polysaccharides, leading to depolymerization and disruption of the foulant matrix. Simultaneously, nitrate- and nitrite-derived reactive species may further promote the destabilization of organic deposits and can also affect the physicochemical properties of the membrane surface. As a result, the adhesion forces between the fouling layer and membrane surface are reduced, facilitating detachment of the cake and gel layers. In addition to the oxidative degradation of foulants, PAW treatment can increase membrane wettability through the introduction of oxygen-containing functional groups, enhancing the surface free energy and reducing the affinity between the membrane surface and organic foulants (Wong et al., 2023; Zhang et al., 2026; Zhou et al., 2020). However, excessive plasma exposure can result in over-oxidation, which can adversely affect membrane performance. Through prolonged oxidative treatment, partial degradation can be induced in the cellulose acetate matrix, altering membrane pore morphology through pore enlargement or collapse, and consequently increasing hydraulic resistance, which explains the observed decline in flux under certain conditions. Although the concentrations of individual RONS were not quantified in the present study, previous studies have identified H_2_O_2_ and nitrate-derived species as major contributors to the cleaning efficacy of PAW. The quantification of key physicochemical parameters, including H_2_O_2_, NO_2_^−^, NO_3_^−^, oxidation–reduction potential, and electrical conductivity, was beyond the scope of the present study. Therefore, the proposed mechanisms underlying PAW-assisted membrane cleaning are based on the established literature and should be considered as plausible mechanistic interpretations rather than definitive conclusions.

A reduction in the distance between the electrodes and the water surface generally enhances the local electric field intensity and plasma density at the plasma-water interface, increasing the reactive species production (Burlica et al., 2006). However, with excessive plasma exposure due to prolonged water activation times or small electrode-to-water surface distance, the elevated concentration of reactive species can adversely affect membrane performance, for instance, on membrane selectivity by retaining too many hydrophilic solutes. Also, increased hydrophilicity may sometimes alter rejection or flux behavior depending on feed composition and membrane pore size. While PAW with moderate reactive species enhances membrane wettability and permeate flux by mitigating hydrophobic fouling, excessive RONS generated under prolonged CP activation or an excessively small electrode-to-water surface distance can increase hydraulic resistance and promote pore blockage. Excessive membrane hydrophilicity can promote the formation of a strongly bound hydration layer on the membrane surface through enhanced water adsorption and retention, acting as an additional mass-transfer resistance, effectively reducing the hydraulic pore diameter and impeding water transport through the membrane. As a result, the hydraulic resistance and permeate flux would be increased and decreased, respectively (Darvish et al., 2020; Fitri & Widiastuti, 2017).

### 3.3 Number of cleaning cycles

The ANOVA of the PAW production parameters on membrane recovery, as evaluated by the number of cleaning cycles, is presented in Table 4. The results indicate that only the electrodes-to-water surface distance (B) had a statistically significant linear effect on the number of cleaning cycles (*P* *<* *0.05*), whereas the PAW activation time (A) was not significant (*P* *>* *0.05*). Moreover, both the interaction term (AB) and the quadratic term of electrode-to-water surface distance (B^2^) significantly affected the number of cleaning cycles (*P* *<* *0.05*). On the other hand, the quadratic term of the PAW activation time (A^2^) showed no significant impact (*P > 0.05*).

**Table 4:** ANOVA for the effect of PAW production conditions on the number of membrane recoveries.

Source	Sum of Squares	df	Mean Square	F Value	P-value
Model	17.72	5	3.54	14.91	0.0050
A (Plasma Time)	1.50	1	1.50	6.31	0.0537
B (Distance)	2.67	1	2.67	11.22	0.0203
AB	2.25	1	2.25	9.46	0.0276
A^2^	0.39	1	0.39	1.66	0.2539
B^2^	11.23	1	11.23	47.23	0.0010
Lack of Fit	1.19	3	0.40	…	…

**Table 5:** Coefficients of regression models for predicting of the pH of PAW, permeate flux after first cleaning process, and the maximum number of cleaning cycles before membrane aging.

Y	$\beta_{0}$	Coefficients of first-order effects		Coefficients of interactions			R^2^
		$\;\beta_{1}$	$\;\beta_{2}$	$\;\beta_{12}$	$\;\beta_{11}$	$\;\beta_{22}$	
Permeate flux kg/ (m^2^.h)	−8.3489	−0.4401	0.8772	0.0099	0.0267	−0.0142	0.928
pH	4.1237	−0.5137	0.0899	0.0016	0.0225	−0.0013	0.987
Maximum number of cleaning cycles	−5.1447	−2.2565	1.0673	0.0375	0.0986	−0.0210	0.937

**Table 6:** The color parameters of apple juice before and after membrane clarification.

Properties		Primary membrane before cleaning		Membrane after first PAW cleaning		Membrane after chemical cleaning	
		Permeate	Retentate	Permeate	Retentate	Permeate	Retentate
Color parameters	L*	87.31 ± 0.24^a^	24.11 ± 0.45^C^	85.64 ± 0.17^a^	25.23 ± 0.42^B^	76.37 ± 0.51^b^	28.41 ± 0.35 ^A^
	a*	3.98 ± 0.12^a^	−4.65 ± 0.11^B^	3.81 ± 0.07^a^	−4.50 ± 0.21^B^	2.17 ± 0.16^b^	−4.12 ± 0.15 ^A^
	b*	5.12 ± 0.10^c^	8.75 ± 0.13^B^	5.49 ± 0.18^b^	8.81 ± 0.15 ^A^	5.89 ± 0.10^a^	8.98 ± 0.09 ^A^

*The same small letter in the same row indicates no significant differences between values in Permeate. **The same capital letter in the same row indicates no significant differences between values in Retentate. (Duncan's test, *P* *<* *0.05*).

As shown in Fig. 2c, the maximum number of cleaning cycles was achieved by applying PAW generated with a 9-min gliding arc plasma activation with a 30 mm distance between the electrodes and the water surface. The number of cleaning cycles is an inherently discrete response variable; however, it was modeled using a second-order polynomial to facilitate comparative optimization of the PAW operating conditions rather than for strict statistical inference. In contrast, the minimum number of cleaning cycles was recorded at a 20 mm electrode-to-water surface distance and 9- and 7-min activation time. The generated RONS impart strong oxidative properties to PAW. Although RONS play a crucial role in the oxidative degradation and removal of membrane foulants, their excessive concentrations resulting from prolonged plasma exposure or high intensity can induce oxidative degradation of polymeric membrane materials, weakening the polymer chains, leading to changes in membrane permeability, selectivity, and mechanical integrity. MCE membranes are especially susceptible to oxidative damage due to the susceptibility of the hydroxyl groups in cellulose to RONS, which can lead to chain scission and membrane deterioration (Duceac et al., 2022; Li et al., 2024; Sołtan et al., 2022).

The proposed PAW production parameters for membrane recovery were identified as a 9 min activation time and a 30 mm electrode-to-water surface distance to achieve the maximum permeate flux and the highest number of cleaning cycles. The close agreement between the predicted and experimental permeate flux values, with a relative deviation of 4.29%, reveals the high accuracy and reliability of the proposed model.

**Fig. 3:**
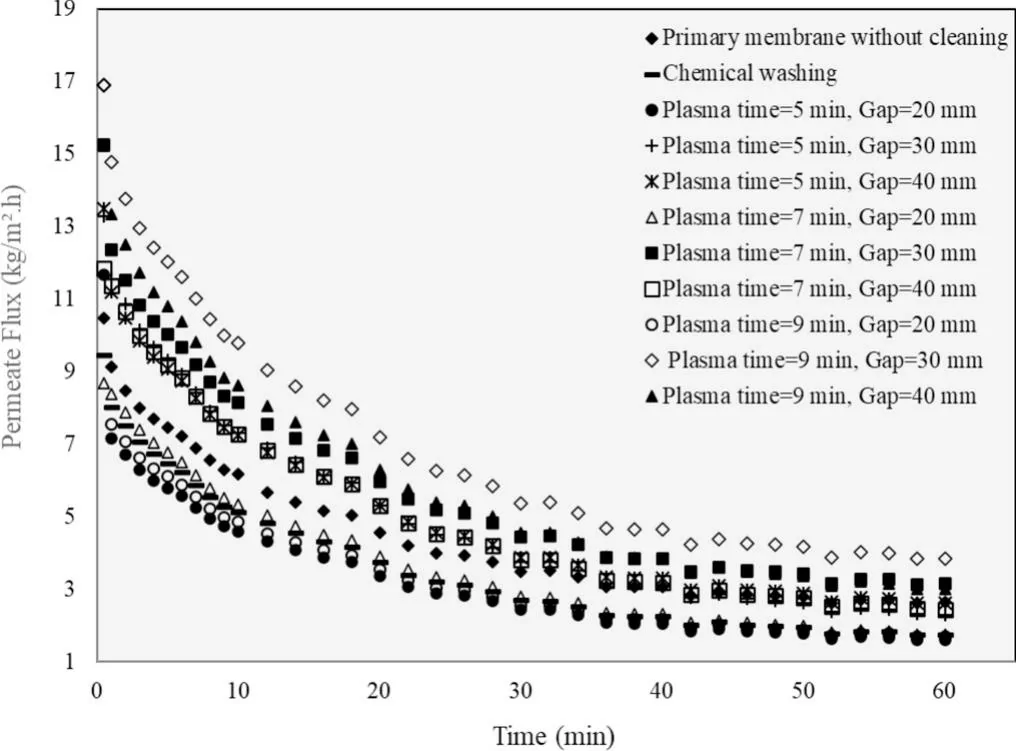
Permeate flux after the first cleaning process of the membrane using PAW produced by different conditions, chemically washed membrane, and primary membrane.

Changes in the permeate flux after the first cleaning process using PAW produced under different operating conditions are presented in Fig. 3. The results demonstrate that membrane recovery by PAW can either enhance or reduce permeate flux during apple juice clarification, depending on the PAW production conditions. As shown in Fig. 3, PAW generated with a 20 mm electrode-to-water distance resulted in a lower permeate flux than that obtained after conventional chemical cleaning. On the contrary, PAW produced under the remaining operating conditions achieved higher permeate fluxes, indicating more effective membrane recovery. The reduction in permeate flux observed at the shortest electrode-to-water surface distance can be related to membrane surface damage and pore blockage, which increased hydraulic resistance. Conversely, PAW generated under moderate plasma conditions can promote effective foulant removal, likely enhancing water transport through the membrane, resulting in higher permeate flux (Yan et al., 2026).

### 3.4 FRR

**Fig. 4:**
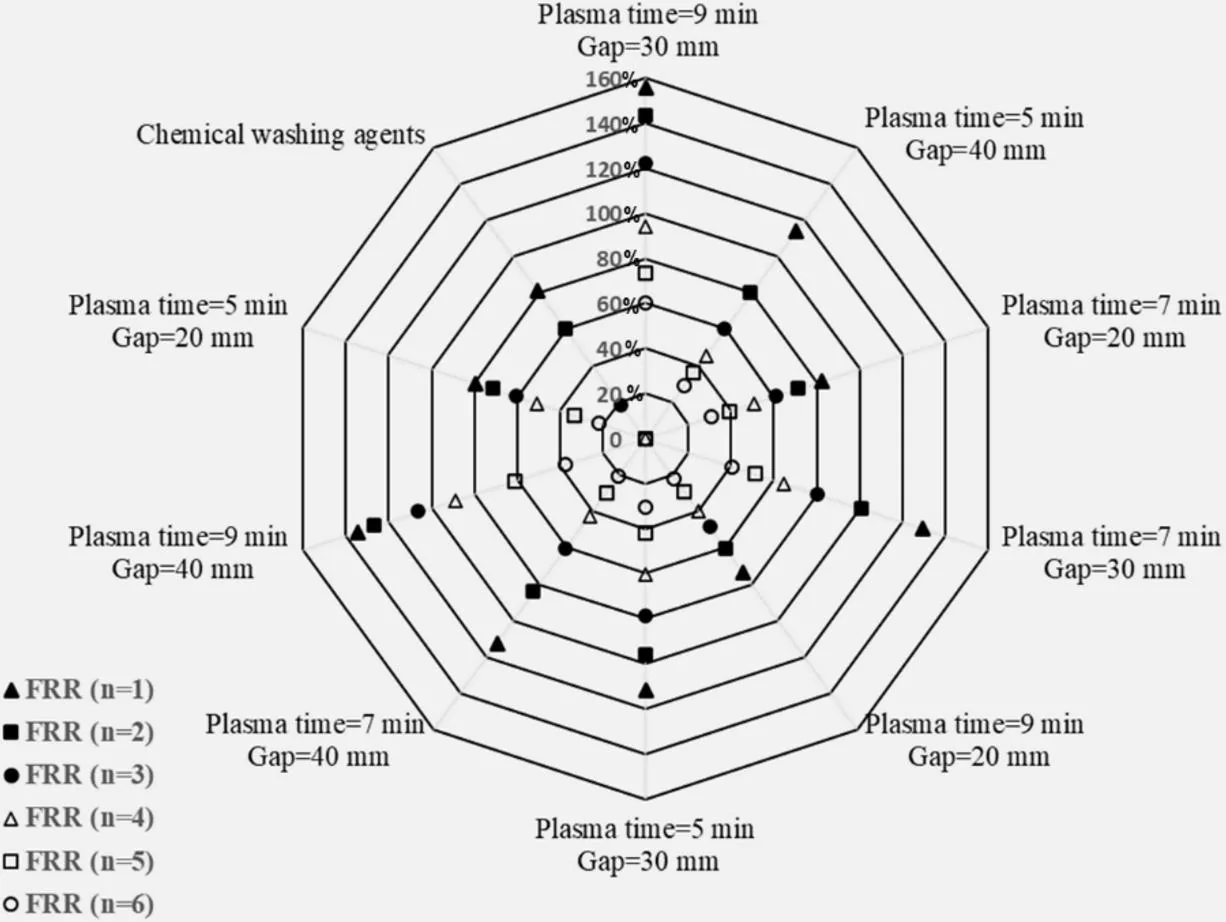
Flux recovery ratio (FRR) after n cleaning processes of the membrane using PAW produced by different conditions and cleaning with chemical agents.

FRR is a key parameter to evaluate the efficiency of membrane cleaning procedures. Higher FRR values indicate more efficient foulant removal and membrane permeability recovery. Accordingly, FRR was employed as a standardized metric to assess the effectiveness of cleaning protocols based on their flux recovery performance (He et al., 2025; Zhang et al., 2023). As shown in Fig. 4, six consecutive cleaning cycles (*n* = 1–6) were investigated for all PAW treatments and the conventional chemical cleaning procedure. The maximum number of cleaning cycles was defined as the cycle at which the FRR decreased below the acceptable threshold value of 60%. In the initial screening experiments, rinsing with untreated water (i.e., without plasma activation) was used as the baseline control, showing no significant recovery of permeate flux and no meaningful improvement in FRR. These findings indicate the insufficiency of hydraulic rinsing alone to remove strongly adhered foulants, suggesting the significant role of irreversible fouling and pore blockage in limiting membrane recovery under water-only cleaning conditions. Therefore, effective membrane regeneration during the microfiltration of apple juice requires either conventional chemical cleaning or green alternatives, such as PAW, to restore membrane performance and maintain sustainable operation. However, the potential presence of residual reactive species following PAW treatment was not investigated in this study. Further research should therefore evaluate residual reactive species and their potential implications for food safety to further validate the applicability of PAW as a membrane cleaning agent in food processing.

As shown in Fig. 4, the FRR of membranes cleaned with PAW progressively decreased with increasing cleaning cycles under all operating conditions. FRR is a critical indicator of membrane cleaning efficiency and operational sustainability, reflecting the ability of the cleaning process to restore membrane permeability after repeated use. In this study, an FRR value below 60% was adopted as an operational threshold to indicate a substantial decline in flux recovery efficiency. This threshold was employed as a practical performance-based criterion to compare the durability and effectiveness of repeated PAW cleaning cycles under different operating conditions, rather than as a definitive indicator of irreversible membrane aging or structural degradation (Asif Khan et al., 2023). Beyond this threshold, further cleaning can result in membrane surface damage and inadequate foulant removal, rendering subsequent cleaning cycles ineffective and potentially detrimental to membrane integrity. Accordingly, an FRR of 60% was adopted as the minimum acceptable flux recovery threshold to define the endpoint of repeated cleaning cycles. Cleaning protocols were optimized to maintain the FRR above this level, ensuring effective fouling control while preserving membrane integrity. Therefore, the response surface results for this variable should be interpreted as a ranking and optimization tool rather than an exact continuous predictive model. The highest FRR was achieved using PAW generated with a 9-min activation time and a 30-mm electrode-to-water surface distance. Under these conditions, the FRR remained above the 60% threshold for up to six consecutive cleaning cycles, indicating superior cleaning stability and sustained membrane performance. In contrast, when PAW was generated using the same activation time with a 20-mm electrode-to-water surface distance, the FRR declined to 60% by the third cleaning cycle, indicating reduced cleaning efficacy. A similar decline was also observed for membranes cleaned using conventional chemical agents, with the FRR also reaching 60% at the third cycle. Such a reduction can be related to progressive membrane damage and incomplete foulant removal during repeated cleaning cycles. The results suggest the PAW generation conditions significantly affect the number of effective cleaning cycles that can be achieved before membrane performance deteriorates (*P* *<* *0.05*).

### 3.5 SEM analysis

**Fig. 5:**
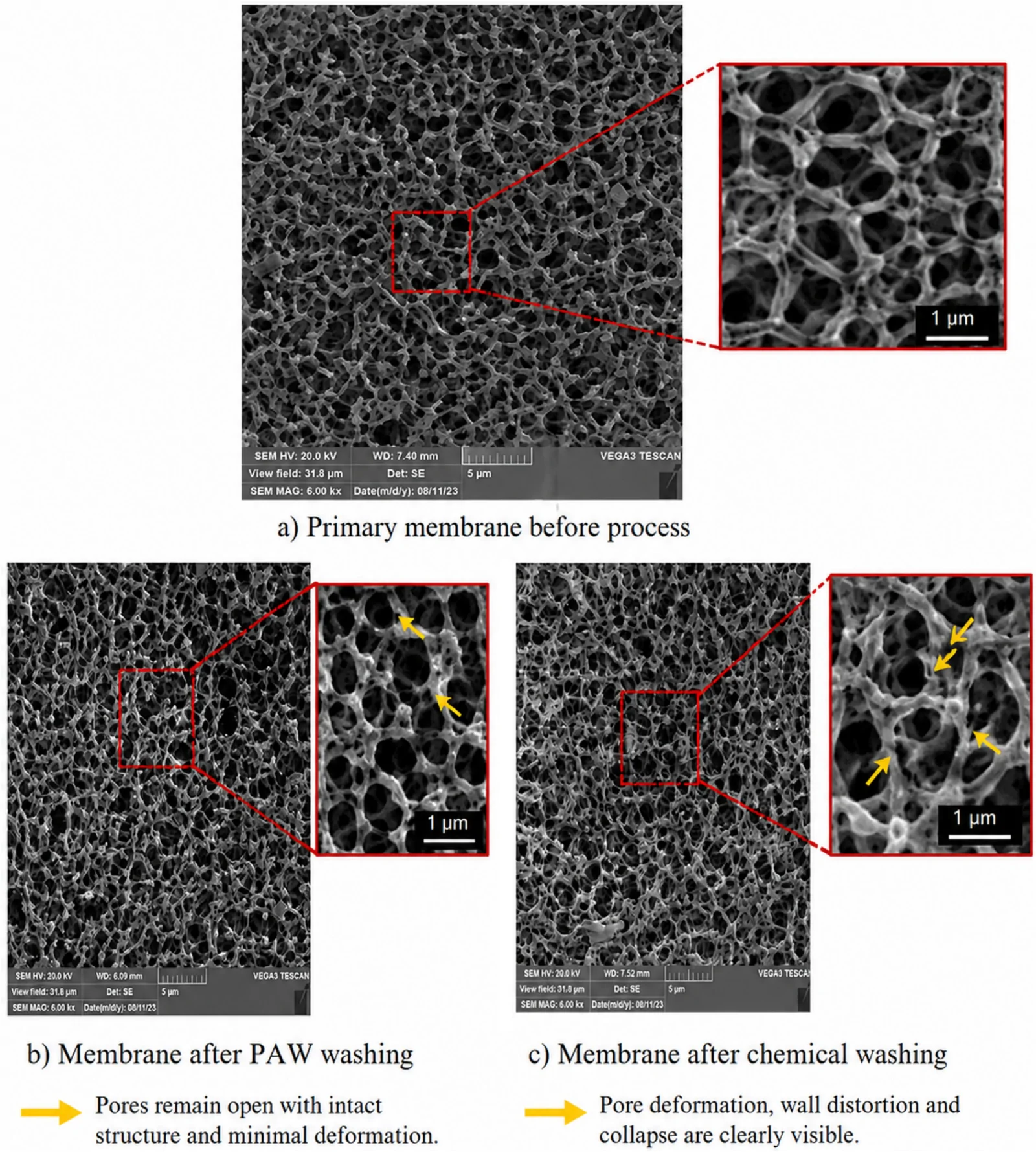
SEM images of membrane cross-section after cleaning the membrane using PAW produced at 9-min water activation time with CP and a 30 mm electrode-to-water surface distance, and cleaning with chemical agents.

SEM images of the primary membrane, the membrane after PAW cleaning, and the membrane after conventional chemical cleaning are presented in Fig. 5. Results demonstrate that the primary MCE membrane exhibited a uniform porous structure with a homogeneous pore size distribution, indicating an intact membrane matrix without visible defects or alterations in its original morphology. Following PAW cleaning, the membrane retained its porous architecture, with only minor changes in pore size and morphology. The pores remained relatively uniform and well-defined, suggesting that PAW cleaning exerted minimal physical or chemical impact on the membrane structure. On the other hand, the membrane subjected to conventional chemical cleaning exhibited noticeable structural deterioration, including irregular and distorted pores, partially collapsed pore walls, and localized erosion, leading to a less uniform porous network and decreased structural integrity that reflect the harsh impact of chemical agents on the cellulose ester membrane. The SEM observations indicate that PAW cleaning better preserved the membrane pore morphology than the applied chemical cleaning protocol. However, due to the two cleaning procedures differing in several operational parameters, including temperature, pH, and chemical chemistry, the observed morphological differences cannot be related exclusively to the cleaning agent. The primary membrane morphology provides a reference for evaluating the structural changes induced by the respective cleaning protocols.

### 3.6 Color properties

The color parameters of the permeates and retentate obtained during clarification using the primary membrane, the PAW-cleaned membrane, and the chemically cleaned membrane are presented in Table 6. Due to the scope of this study, juice quality evaluation was limited to color measurements, while other quality attributes were not investigated. Compared with PAW cleaning, conventional chemical cleaning resulted in a significant decrease in the L* (lightness) and a* (red-green) values of the permeate, while these parameters increased in the retentate (*P* *<* *0.05*). Such changes indicate that the chemical cleaning protocol affected the color properties, potentially due to residual cleaning chemicals, modifications to membrane surface properties, or differences in membrane separation performance following repeated cleaning. In contrast, PAW cleaning resulted in no significant changes in L* and a* values of either the permeate or the retentate (*P* *>* *0.05)*, suggesting the minimal impact of this cleaning approach on the color characteristics. Additionally, the b* (yellow-blue) value increased in the permeate and decreased in the retentate following both PAW and conventional chemical cleaning. However, such changes were more pronounced after chemical cleaning. This can be attributed to the differences in fouling removal efficiency or alterations in membrane structure induced by the respective cleaning protocols. Structural damage to membrane pores can decline membrane selectivity, leading to changes in the transport and retention of pigments, colloidal particles, and other suspended macromolecules, which can consequently affect juice color intensity, hue, turbidity, and optical clarity (Katibi et al., 2023; Roberge et al., 2022).

## 4 Conclusion

This study demonstrates that gliding arc plasma-generated PAW is an effective and sustainable cleaning agent for restoring the performance of fouled plasma-modified MCE microfiltration membranes used in apple juice clarification. The optimal PAW generation conditions (9 min activation time and a 30 mm electrode-to-water surface distance) significantly improved permeate flux and increased the number of effective cleaning cycles, as confirmed by RSM. Compared with conventional chemical cleaning, PAW achieved superior membrane recovery, greater flux stability, and better preservation of membrane integrity, as supported by 10.13039/100014281SEM analysis, while maintaining the color properties of both the clarified juice and the retentate. These findings suggest PAW as a promising alternative to conventional chemical cleaning, enabling sustainable membrane regeneration through prolonged membrane lifetime, reduced chemical consumption, and lower operating costs. Despite its promising findings, this study was primarily process-oriented, which focused on the mechanisms and process conditions of PAW optimization for membrane cleaning and recovery. Therefore, comprehensive evaluations of the physicochemical, nutritional, and microbiological attributes of the clarified apple juice can be investigated in future studies. Future investigations can also explore the applicability of the optimized PAW cleaning strategy to other juices and membrane-based separation procedures. Furthermore, given the emerging nature of CP-based technologies, future studies are warranted to focus more on the potential impacts of residual reactive species associated with CP-derived treatments, such as PAW, to support their safe and broader applications in the food industry.

## CRediT authorship contribution statement

**Najmeh Ghahari:** Writing – review & editing, Writing – original draft, Validation, Software, Resources, Investigation, Data curation. **Yousef Ramezan:** Writing – review & editing, Writing – original draft, Validation, Project administration, Methodology, Conceptualization. **Hossein Mirsaeedghazi:** Validation, Project administration, Conceptualization. **Alireza Faraji:** Resources, Methodology. **Shahrooz Saviz:** Resources, Methodology.

## Declaration of competing interest

I wish to confirm that there are no known conflicts of interest associated with this publication and there has been no significant financial support for this work that could have influenced its outcome.

## Data Availability

Data will be made available on request.
